# A FASN-TGF-β1-FASN regulatory loop contributes to high EMT/metastatic potential of cisplatin-resistant non-small cell lung cancer

**DOI:** 10.18632/oncotarget.10837

**Published:** 2016-07-25

**Authors:** Li Yang, Fuquan Zhang, Xin Wang, Ying Tsai, Kuang-Hsiang Chuang, Peter C. Keng, Soo Ok Lee, Yuhchyau Chen

**Affiliations:** ^1^ Department of Radiation Oncology, University of Rochester School of Medicine and Dentistry, Rochester, NY 14642, USA

**Keywords:** non-small cell lung cancer, EMT, cisplatin-resistance, fatty acid synthase (FASN), TGF-β1

## Abstract

Cisplatin-resistant A549CisR and H157CisR cell lines were developed by treating parental A549 (A549P) and H157 (H157P) cells. These cisplatin-resistant cells showed slight growth retardation, but exhibited higher epithelial-mesenchymal transition (EMT) and increased metastatic potential compared to parental cells. We observed a highly up-regulated fatty acid synthase (FASN) level in A549CisR and H157CisR cells compared to parental cells and the up-regulation of FASN was also detected in A549P and H157P cells after short time treatment with cisplatin, suggesting that the high level of FASN in cisplatin-resistant cells may be from the accumulated cellular responses during cisplatin-resistance developmental process. We next investigated whether the inhibition of FASN by using a specific FASN inhibitor, cerulenin, can influence growth and EMT/metastatic potential of A549CisR and H157CisR cells. There was slight growth inhibition, but significantly reduced EMT/metastatic potential in cisplatin-resistant cells upon inhibitor treatment. The *in vitro* result was further investigated in orthotopic xenograft mouse models established with luciferase-tagged H157P and H157CisR cells. Mice were injected with cerulenin or vehicle after tumors were developed. No significant tumor regression was detected at the end of cerulenin treatment, but IHC staining showed higher expression of EMT/metastasis markers in H157CisR cell-derived tumors than H157P cell-derived tumors, and showed dramatic reduction of these markers in tumor tissues of cerulenin-treated mice, confirming the *in vitro* results. In mechanism dissection studies, we revealed the existence of the FASN-TGF-β1-FASN positive loop in A549CisR and H157CisR cells, but not in parental cells, which is believed to augment the FASN function in cisplatin-resistant cells.

## INTRODUCTION

Lung cancer is a predominant cause of cancer death in both men and women [1]. Lung cancer is heterogeneous and histologically divided into two types: small cell lung carcinoma (SCLC) and non-small cell lung carcinoma (NSCLC). NSCLC comprises 85% of lung cancer cases [2] and constitutes a heterogeneous population of squamous, adenocarcinoma, and large cell carcinomas [1]. Platinum-based drugs, particularly cis-diammine-dichloroplatinum (II) (cisplatin, DDP), are used in the treatment of many cancers, including lung cancer. Cisplatin treatment usually shows initial success, but patients often develop chemoresistance later on [3, 4].

Cancer cells need more energy than normal cells, and elevated fatty acid metabolism is important in providing lipids for membrane formation and energy production. Fatty acid synthase (E.C. 2.3.1.85; FASN) is a multifunctional enzyme system that catalyzes the formation of fatty acids from acetyl-CoA, malonyl-CoA, and NADPH, and plays a central role in lipid biosynthesis [5]. Accumulating evidence shows elevated expression/activity of FASN in many types of cancers [6–8]. Implications of FASN in tumor growth in various cancers have been suggested [9–11]. Small molecule FASN inhibitors such as cerulenin [12, 13], C75 [14], and orlistat [15] have been shown to induce apoptosis in several cancer cell lines and induce tumor growth delay.

The role of FASN in epithelial-mesenchymal transition (EMT) has also been investigated. Li *et al* [16] reported FASN mediation of EMT in breast cancer cells, and Hung *et al* [17] showed that inhibition of FASN abrogated the EMT process in breast cancer. However, a contradictory report stated that FASN knockdown enhanced EMT in lung cancer cells [18].

The EMT process is also thought to be correlated with drug resistance development. Piskareva *et al* [19] observed EMT during drug resistance development in neuroblastoma and Liu *et al* [20] also showed a functional link between EMT phenotype and drug resistance. In addition, the role of FASN in triggering drug resistance via regulation of molecules involved in apoptosis and DNA repair pathways has also been suggested [21]. Likewise, specific inhibition of FASN was shown to sensitize cisplatin-resistant breast cancer cells to cisplatin [22].

In this study, we revealed the role of FASN in mediating EMT/metastasis increase in cisplatin-resistant lung cancer, which will have great clinical significance as increased invasive features of cisplatin-resistance cells have been reported [23, 24]. We further elucidated molecular mechanisms to govern this regulation.

## RESULTS

### Growth is retarded, but EMT/migration potential is higher in cisplatin-resistant NSCLC cells than parental cells

**Figure 1:**
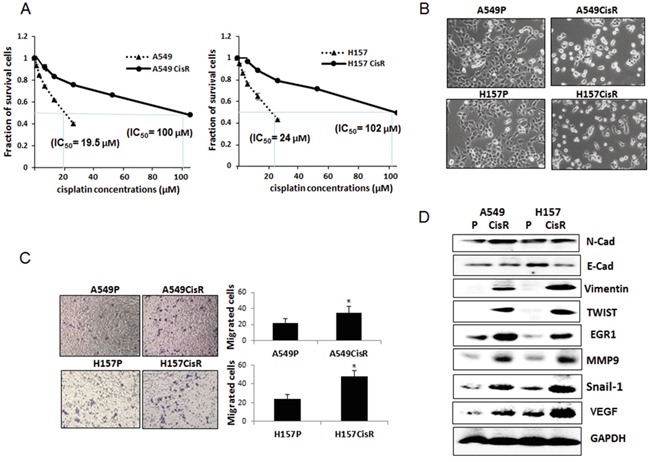
EMT and metastatic potential were increased in cisplatin-resistant NSCLC cells compared to parental cells **A.** Cytotoxicity test of A549P/H157CisR and H157P/H157CisR cells against cisplatin treatment. Cisplatin-resistant cells were obtained by continuous treatment of cells with increasing dose of cisplatin for 6 months. Cisplatin cytotoxicities of A549P/H157CisR and H157P/H157CisR cells were analyzed in the presence of various concentrations of cisplatin in MTT assay. **B.** Morphology test. A549P/H157CisR and H157P/H157CisR cells (1 × 10^3^) were seeded and morphology was observed under microscope. **C.** Migration test. Cells (A549P/A549CisR and H157P/H157CisR, 1 × 10^4^) were placed in upper chamber of transwell plates (8 μm pore) and migrated cells to lower chamber were counted under microscope after crystal violet staining at the end of 24 hours of incubation. Quantitation shown on right. **D.** Western blot analysis showing an increase in EMT/metastasis markers in cisplatin-resistant cells compare to parental cells. Cell extracts were obtained from A549P/A549CisR and H157P/H157CisR cells and Western blot analyses were performed using antibodies against indicated molecules. *p<0.05, **p<0.01.

We developed two cisplatin-resistant NSCLC cell lines, A549CisR and H157CisR, by treating A549P and H157P cells with an increasing dose of cisplatin over 6 months [26]. These cells showed about 5 times higher IC_50_ values than parental cells (Figure 1A).

We observed slight growth retardation of A549CisR and H157CisR cells compared to their parental cells (data not shown). However, significantly increased EMT/metastatic potential was observed in A549CisR and H157CisR cells compared to their parental cells. First, morphology studies showed that A549CisR and H157CisR cells exhibit mesenchymal phenotype (Figure 1B). Consistently, migration assays using transwell plates showed increased migration abilities of A549CisR and H157CisR cells compared to parental cells (Figure 1C). When expression of the EMT markers, N-cadherin (N-cad), E-cad, vimentin, Snail-1, and TWIST, and the metastasis-related molecules matrix metalloproteinase 9 (MMP9), vascular endothelial growth factor (VEGF), and early growth response 1 (EGR1) were investigated, up-regulation of these markers were also observed in A549CisR and H157CisR cells compared to parental cells (Figure 1D). Meanwhile, down-regulation of E-Cad, which represents an indication of EMT increase, was shown in A549CisR and H157CisR cells compared to parental cells (Figure 1D).

### Up-regulation of FASN in cisplatin-resistant cells and short-time cisplatin-treated parental cells

**Figure 2:**
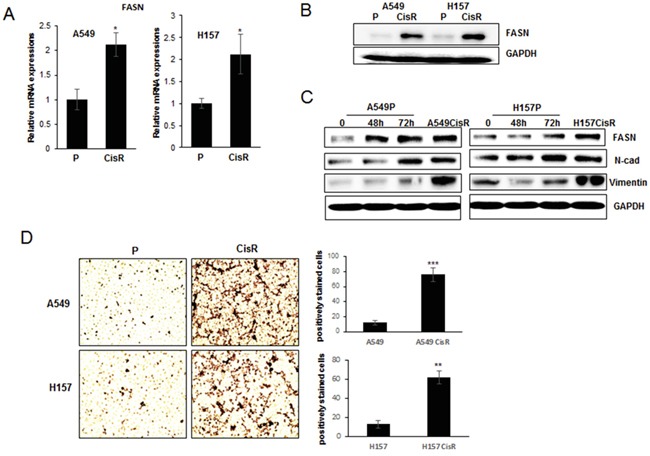
Increased FASN expressions in cisplatin-resistant NSCLC cells compared to parental cells **A.** qPCR analysis of FASN in A549P/A549CisR and H157P/H157CisR cells. **B.** Western blot analysis of FASN in A549P/A549CisR and H157P/H157CisR cells. **C.** Western blot analysis of FASN in A549P and H157P cells upon cisplatin treatment. Cells were treated with 5 μM cisplatin for indicated time and cell extracts obtained were used in analyses. **D.** Oil Red staining. Equal number (1 × 10^3^) of A549P/A549CisR and H157P/H157CisR cells was plated in 24 well plates. After settlement, cells were stained with Oil Red O working solution for 15 min at room temperature, and washed with running tap water to remove unbound staining. The positively stained cells per phase were counted under a microscope. Quantitation shown on right. *p<0.05, **p<0.01, ***p<0.001.

There were significantly higher FASN levels in A549CisR and H157CisR cells than in parental cells (Figure 2A, qPCR data, Figure 2B, Western blot data). To determine whether the increased levels of FASN in cisplatin-resistant cells is due to accumulated effects of cisplatin treatment, the FASN levels in A549P and H157P cells after short-term cisplatin treatment were also investigated. As shown in Figure 2C, we found up-regulation of FASN in A549P and H157P cells when they were treated with cisplatin (5 μM) for 48 to 72 hours (Figure 2C). Increase of two randomly selected EMT markers, N-cad and vimentin, was shown in these cells after cisplatin treatment (Figure 2C).

Oil Red staining, which is the method used for quantitation of fatty acids [27], was applied. In this staining, significantly higher numbers of Oil Red positively stained cells were detected in A549CisR and H157CisR cells than in parental cells (Figure 2D), suggesting higher fatty acid metabolism in cisplatin-resistant cells and supporting the results showing increased FASN expression in these cells.

### FASN inhibition resulted in slight growth reduction, and significant reduction in EMT/metastatic potential of cisplatin-resistant cells

**Figure 3:**
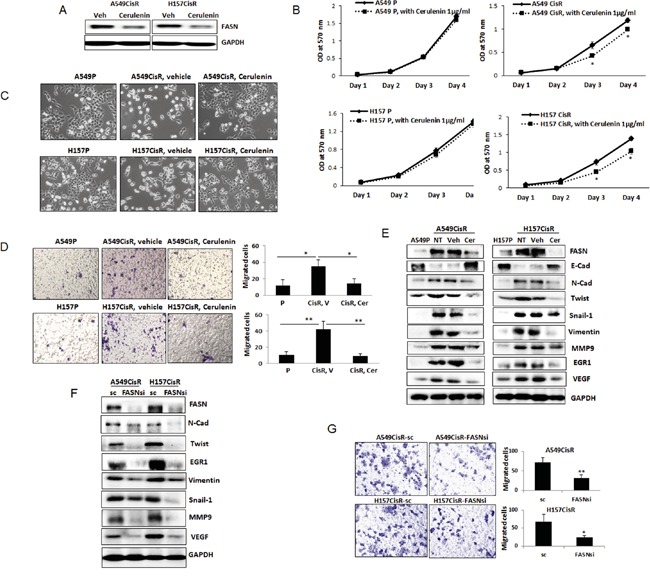
Inhibition of FASN reduced EMT/metastasis potential of cisplatin-resistant cells **A.** Western blot analyses of FASN in A549CisR and H157CisR cells upon cerulenin (1 μg/ml, 48 hours) treatment. **B.** Growth assay. A549P and H157P and A549CisR and H157CisR (1 × 10^3^) were plated in 96 well plates, and the growth of cells in presence of cerulenin (1 μg/ml) or vehicle (control) was analyzed at each day in MTT assays. **C.** Morphology test. A549P, H157P, A549CisR and H157CisR cells were seeded. A549CisR and H157CisR cells were treated with cerulenin (1 μg/ml) or vehicle (control) for 48 hours and cell morphology was observed under a microscope. **D.** Migration test. A549P, H157P, A549CisR and H157CisR cells were seeded. A549CisR and H157CisR cells (1 × 10^4^) were treated with cerulenin (1 μg/ml) or vehicle (control) for 48 hours. Cells were then placed in upper chamber of transwell plates and migrated cells to the lower chamber at the end of 24 hours incubation were counted under a microscope after crystal violet staining. Quantitation shown on right. **E.** Western blot analyses of EMT/metastasis markers. Cell extracts were obtained from parental cells and cerulenin (or vehicle)-treated A549CisR and H157CisR cells and Western blot analyses were performed using antibodies against indicated molecules. **F.** Western blot analyses of EMT/metastasis markers in A549CisR-FASNsi/sc and H157CisRCisR-FASNsi/sc cell sets. Cell extracts were obtained and Western blot analyses were performed using antibodies against indicated molecules. **G.** Migration test. A549CisR-FASNsi/sc and H157CisRCisR-FASNsi/sc cells were placed in upper chamber of transwell plates and migrated cells to the lower chamber at the end of 24 hours incubation were counted under a microscope after crystal violet staining. Quantitation shown on right. *p<0.05, **p<0.01.

To test whether FASN inhibition can affect the growth and EMT/metastatic potential of cisplatin-resistant lung cancer cells, a FASN inhibitor cerulenin was added to the A549CisR and H157CisR cell culture. As shown in Figure 3A, this drug treatment reduced the FASN level, which is consistent with the previous report [28]. In a growth test, slightly reduced growth of A549CisR and H157CisR cells was observed after cerulenin treatment, but no difference was detected in parental cells (Figure 3B). More interestingly, we observed morphology changes (Figure 3C), significantly reduced migration potential (Figure 3D), and significantly reduced expression of the EMT/metastasis markers (Figure 3E) in A549CisR and H157CisR cells after cerulenin treatment.

The FASN knocked down A549CisR and H157CisR cells were also developed (A549CisR-FASNsi and H157CisR-FASNsi) together with scramble (sc) control cells. When expression of EMT/metastasis markers in these cells were investigated, significantly reduced expression of EMT/metastasis markers were observed in FASNsi cells compared to sc control cells (Figure 3F). In addition, the FASNsi cells exhibited significantly reduced migration potential than sc control cells (Figure 3G). These results suggest that FASN plays a critical role in mediating EMT/metastasis increase in cisplatin-resistant NSCLC cells.

### *In vivo* studies using orthotopic xenograft mouse models confirmed *in vitro* results

**Figure 4:**
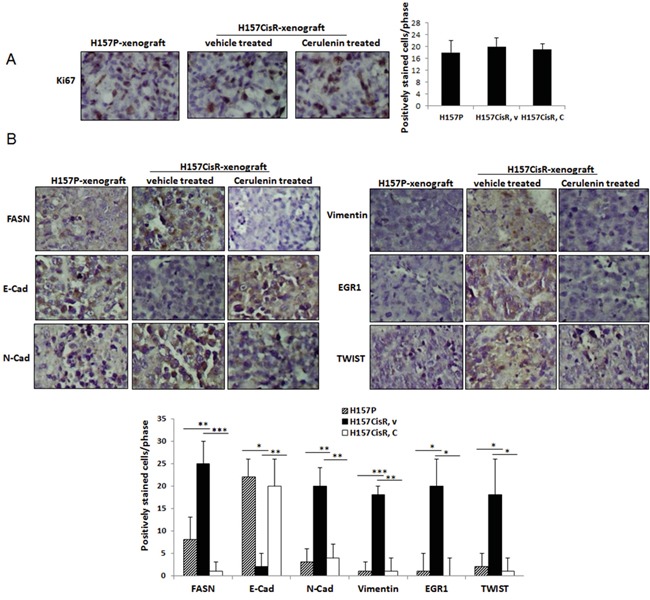
*In vivo* mice studies **A, B.** IHC staining of tumor tissues using antibodies of Ki67 (A) and EMT/metastasis markers (B). Mice (H157CisR cell-derived xenograft) were sacrificed 3 days at the completion of cerulenin (or vehicle) treatment (H157P cell-derived xenografts did not receive treatment) and tumor tissues were obtained. The processed tumor tissues were cut into 5 μm, and stained with antibodies of FASN and EMT/metastasis markers. For Ki67 staining, tissues were subjected to antigen retrieval before staining. Magnification, x 100. Quantitation shown below. *p<0.05, **p<0.01, ***p<0.001.

To confirm the *in vitro* results showing FASN contribution in mediating EMT/metastasis increase in cisplatin-resistant cells, *in vivo* mice studies were performed. Orthotopic xenograft mouse models were developed [29] by injecting luciferase-tagged H157P (n=6) and H157CisR cells (n=14). Tumor development was monitored once a week by *In Vivo* Imaging System (IVIS) with luciferin injection. When luminescence reached to 5 × 10^5^ to 1 × 10^6^ radiance (p/sec/cm^2^/sr), which corresponds to tumor size of 300-400 mm^3^ (based on our previous unreported results), H157CisR cells-inoculated mice were divided into two groups. The test group mice (n=7) were i.p. injected with cerulenin (15 mg/kg) and the control group mice (n=7) were injected with vehicle (20% DMSO) for 9 days. Three days after the last injection of cerulenin/vehicle, tumor growth and metastasis in both groups of mice were compared, but no obvious changes in tumor size were observed when judged from the luminescence (Supplementary Figure 1). When Ki67 IHC staining was performed using tumor tissues obtained from the H157P cell-derived and H157CisR cell-derived xenografts (both cerulenin and vehicle treated), no significant difference was consistently detected (Figure 4A), suggesting that tumor growth was not significantly influenced by this drug treatment.

IHC staining was further performed using tumor tissues obtained from the H157P cell-derived and H157CisR cell-derived xenografts, vehicle or cerulenin-treated. First, an increased FASN level in tumor tissues of H157CisR cell-derived xenografts were detected compared to tissues of H157P cell-derived xenografts, confirming the *in vitro* result showing an increased FASN level in cisplatin-resistant cells compared to parental cells. More importantly, we found the FASN positive-stained cells were dramatically reduced in tumor tissues obtained from the cerulenin-injected mice compared to the vehicle-injected control mice (Figure 4B).

The IHC staining using antibodies of EMT/metastasis markers (N-Cad, E-Cad, vimentin, TWIST, and EGR1) were also performed. We detected higher positive staining with N-Cad, vimentin, TWIST, and EGR1 in tissues of H157CisR cells-derived xenografts compared to tissues of H157P cells-derived xenografts and found these positive signals were reduced in tissues of the cerulenin-treated mice compared with vehicle-treated mice. The E-cad staining showed the opposite tendency, which also indicates increase of EMT in H157CisR cell-derived tumors and the cerulenin effect in reversing the EMT process in these tumors (Figure 4B).

### TGF-β1 is an important FASN downstream signaling that mediates EMT/meta increase in cisplatin-resistant cells

**Figure 5:**
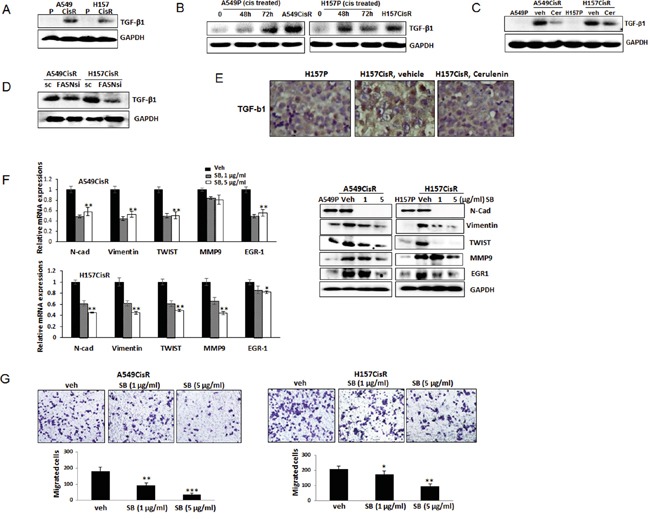
TGF-β1 is FASN downstream signaling that is important in mediating EMT/metastasis increase of cisplatin-resistant NSCLC cells **A.** Western blot analysis showing TGF-β1 levels in A549P/A549CisR and H157P/H157CisR cells. **B.** Western blot analysis showing TGF-β1 increased expression in A549P and H157P cells treated with cisplatin at 5 μM for 48 hours or 72 hours. **C.** Western blot analysis showing TGF-β1 level decrease in A549CisR and H157CisR cells with cerulenin treatment (1 μg/ml, 48 hours). **D.** Western blot analysis showing TGF-β1 levels in A549CisR-FASNsi/sc and H157CisR-FASNsi/sc cells. **E.** TGF-β1 IHC staining of tumor tissues obtained in mice studies. **F.** qPCR (left) and Western blot (right) analyses showing expression of the EMT/metastasis markers in A549P/A549CisR and H157P/H157CisR cells after treatment with either vehicle or the TGF-β1 inhibitor, SB525334 (1 and 5 μg/ml) for 48 hours. **G.** Migration assay using A549CisR and H157CisR cells pre-treated with either vehicle or the TGF-β1 inhibitor, SB525334 (1 and 5 μg/ml, 48 hrs). *p<0.05, **p<0.01, ***p<0.001.

In mechanism dissection studies, we investigated which molecule is the FASN downstream molecule that can mediate EMT/metastasis increase in cisplatin-resistant lung cancer cells. We first investigated TGF-β1 expression in A549CisR and H157CisR cells vs. parental cells as this molecule was reported as the major inducer of EMT in lung cancer [30]. As shown in Figure 5A, higher TGF-β1 levels were detected in A549CisR and H157CisR cells compared to parental cells, and the TGF-β1 up-regulation was also detected in A549P and H157P cells when treated with cisplatin (Figure 5B). In addition, the TGF-β1 levels in A549CisR and H157CisR cells were reduced when cells were treated with the FASN inhibitor, cerulenin (Figure 5C). The decreased expression of TGF-β1 was also detected in A549CisR-FASNsi and H157CisR-FASNsi cells compared to sc control cells (Figure 5D). We also detected a higher number of positively stained cells with TGF-β1 in tissues of H157CisR cell-derived xenografts compared to H157P cell-derived tumor tissues, and the positively stained cell numbers were decreased in cerulenin-treated mice tumor tissues compared to the vehicle-treated mice tissues (Figure 5E).

We then treated A549CisR and H157CisR cells with the specific inhibitor of TGF-β1 signaling, SB525334, and tested whether inhibiting TGF-β1 signaling can inhibit EMT/metastatic potential of cisplatin-resistant cells. When this inhibitor was added into the A549CisR and H157CisR cell cultures, reduced expression of EMT/metastasis markers were detected (Figure 5F left panel, qPCR data; right panel, Western blot data). Reduced migration abilities of A549CisR and H157CisR cells were also observed when these cells were incubated with the TGF-β1 inhibitor (Figure 5G). These results suggest that TGF-β1 may be the FASN downstream molecule that mediates EMT/metastasis increase in cisplatin-resistant cells.

### The TGF-β1-FASN-TGF-β1 positive loop exists in A549CisR and H157CisR cells, but not in parental cells

**Figure 6:**
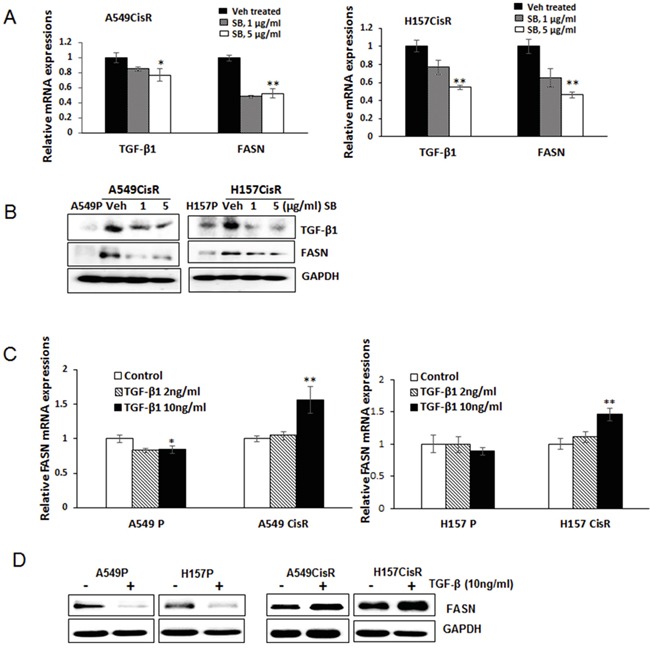
TGF-β1 induces FASN in cisplatin-resistant lung cancer cells **A, B.** qPCR (A) and Western blot (B) analysis showing TGF-β1 and FASN mRNA (A) and protein (B) levels in A549CisR and H157CisR cells upon treatment with either vehicle or the TGF-β1 inhibitor SB525334 (SB) for 48 hours. **C, D.** qPCR (C) and Western blot (D) analysis showing FASN mRNA and protein levels in A549P/A549CisR and H157P/H157CisR cells after culturing with hrTGF-β1 (2 and 10 ng/ml) (24 hours of incubation for mRNA level study and 72 hours of incubation for protein analysis). *p<0.05, **p<0.01.

Interestingly, it was further elucidated that the FASN level was down-regulated in A549CisR and H157CisR cells when treated with the TGFβ-1 inhibitor SB525334 (Figure 6A, qPCR results; Figure 6B, Western blot results). The TGF-β1 regulation of FASN was further confirmed in experiments using the human recombinant TGF-β1 (hrTGF-β1). When the hrTGF-β1was added into the A549CisR and H157CisR cell culture, up-regulation of FASN was detected in these cells, but such regulation was not detected in A549P and H157P cells (Figure 6C, qPCR results; Figure 6D, Western blot results). Together, the results suggest an existence of a positive loop of TGF-β1-FASN-TGF-β1 selectively in A549CisR and H157CisR cells and this regulation may be important in mediating EMT/metastasis increase in cisplatin-resistant lung cancer.

## DISCUSSION

In this study, we demonstrated increased FASN expression in cisplatin-resistant NSCLC cells compared to parental cells. This result is similar to the previous report showing the increased FASN in cisplatin-resistant ovarian cancer cells [28]. We also found this increased FASN level was responsible for the increased EMT and metastatic potential of cisplatin-resistant cells. The implication of FASN in increasing EMT and metastasis of various types of cancer cells has been reported previously [31, 32], but its role in EMT/metastasis increase in cisplatin-resistant lung cancer cells has not been addressed.

It has been controversial regarding which process occurs first, EMT or metabolic reprogramming. Some reports suggest metabolic reprogramming takes place first and that consequently induces EMT/metastasis increase, yet other reports suggest that EMT drives the metabolic changes [33, 34]. It may be also interesting to know whether the FASN-mediated EMT is critical in developing cisplatin-resistance in lung cancer or, on the contrary, cisplatin-resistance will lead to EMT by reprogramming of lipids and up-regulating the FASN level in the cells. Our data showed that the treatment of cisplatin-resistant cells with the FASN inhibitor blocked EMT/metastasis increase, which led us to speculate that the lipid metabolism increase occurs prior to induction of EMT/metastasis. However, there might be a positive feedback control, so more studies are needed to confirm.

It has also been controversial whether EMT leads to metastasis increase or these two processes occur independently. It has been believed that EMT is an essential process in progressing tumor cells into metastasis, but some report suggests that EMT is not the driving force of metastasis [35]. In our studies, whether metastasis increase is a consequence of FASN-induced EMT increase, or that FASN can up-regulate the metastasis-associated molecules independently, is not clear. Further studies are needed.

In this study, we used the FASN specific inhibitor cerulenin. Various concentrations of cerulenin have been applied in *in vitro* and *in vivo* experiments studying different types of cancers, so we performed initial growth and migration tests using different concentrations of cerulenin. We determined that 1 μg/ml of cerulenin is the optimum concentration exhibiting the best inhibitory effects on parental and cisplatin-resistant cells without resulting in cell death (data not shown).

In mice studies, we could not observe obvious metastasis difference among 3 subject groups because tumors attained a large size quickly, although we observed metastasis development in several mice. However, we could clearly detect EMT/metastasis marker differences, indicating EMT/metastatic potential between parental and cisplatin-resistant cell-derived xenografts and between cerulenin and vehicle-treated mice. Further experimentation is necessary to prove whether the difference in this EMT/metastatic potential will indeed lead to a difference in metastasis development. It may be possible to develop mouse models injected with a smaller number of cells and waiting a longer time for metastasis development.

In the mechanism dissection studies, we demonstrated that the FASN effect on increasing EMT/metastasis in cisplatin-resistant cells is through up-regulation of the TGF-β1 signaling. The TGF-β1-induced EMT and increased invasion in various types of cancers has previously been suggested [36]. In this study, we found a FASN-TGFβ1-FASN positive feedback loop in cisplatin-resistant lung cancer cells, but not in parental cells. It was found that TGF-β1 regulates FASN in a different way in cisplatin-resistant cells vs. parental cells; positive regulation was observed in cisplatin-resistant cells, but negative regulation was detected in parental cells. The data showing negative regulation of TGF-β1 on FASN expression in A549P and H157P cells is consistent with a recent report by Jiang *et al* [18]; however, positive regulation of TGF-β1 on FASN expression in cisplatin-resistant cells has never been shown before. This is a novel, interesting discovery and indicates a critical implication of the FASN-TGFβ1-FASN feedback loop in mediating EMT/metastasis increase in cisplatin-resistant lung cancer. To prove the FASN control of TGFβ1 and TGF-β1 control of FASN at the transcriptional levels, further experiments are necessary.

In mechanism studies, we found EGR1 is one of the critical downstream molecules controlled by the FASN-TGFβ1-FASN loop in cisplatin-resistant cells. Although there have been controversial results regarding the role of EGR1 in metastasis control, accumulating evidence shows the positive role of EGR1 in cancer progression and metastasis. In particular, EGR1 is involved in TGF-β1-induced EMT process of NSCLC cells [37]. In this study, we also confirmed that this molecule is induced by TGF-β1 and induces EMT/metastasis increase in cisplatin-resistant NSCLC cells. VEGF is known as a metastasis marker [38], but its implication in the induction of the EMT process has also been suggested [16, 39]. Consistently, we observed that VEGF is another TGF-β1 downstream molecule in mediating EMT/metastasis increase in cisplatin-resistant lung cancer.

Taken together, we suggest that targeting the FASN molecule or TGF-β1 signaling would inhibit EMT/metastasis of cisplatin-resistant lung cancer. In preclinical studies, it was shown that the FASN inhibitor was effective in inducing apoptosis of several tumor cell lines and in reducing tumor growth in several cancer xenograft models [8, 12, 13, 15], but its effect in inhibiting EMT/metastasis has not been extensively studied in animal models. In this study, we demonstrated in our mouse model that targeting FASN can impair EMT/metastasis increase in cisplatin-resistant cells. However, the use of the FASN inhibitor is limited due to side effects such as weight loss [40]. Therefore, the use of the TGF-β1 inhibitor may be an alternative way to target FASN, although complexities of using different types of TGF-β signaling have also been reported [41]. The application of these strategies in clinics may be challenging at this point in time, thus further studies on development of cisplatin-resistant lung tumor mouse models, testing effects of the FASN or TGF-β1 inhibitors, and determination of effective dose and timing in these models are essential.

## MATERIALS AND METHODS

### Cell culture

A549 and H157 cell lines were purchased from the American Type Culture Collection (ATCC, Manassas, VA) and cultured in RPMI 1640 containing 10% FBS. All cells were maintained in a humidified 5% CO_2_ environment at 37°C. For inhibition studies, cerulenin (1 μg/ml, Sigma-Aldrich) and SB525334 (1 μM and 5 μM) that inhibits the FASN and TGF-β1 signaling were added into the culture. Human rTGF-β1 (R&D Systems, Minneapolis, MN) was added into cell culture when necessary.

### Development of cisplatin-resistant cell lines

Parental A549 (A549P) and H157 (H157P) cells were continuously treated with a gradually increased dose of cisplatin for 6 months according to the method described by Barr *et al*. [25] Briefly, cells were treated with 1 μM cisplatin for 72 hours and allowed to recover for the following 72 hours. After repeating one more cycle at 1 μM cisplatin concentration, the cells were then treated with 2 μM cisplatin in the following two cycles. This procedure was conducted repeatedly with increasing cisplatin concentration up to 30 μM. During the cisplatin-resistance induction procedure, the IC_50_ values of every 5 passages were determined and compared with those of the parental cells until the increased IC_50_ value remained unchanged. The cisplatin-resistant cell lines obtained by this method were maintained in growth media containing 10 μM cisplatin.

### Cisplatin-cytotoxicity test

Cisplatin-cytotoxicity was analyzed by MTT (3-[4,5-dimethylthiazol-2-yl]-2,5-diphenyltetrazolium bromide, 5 mg/ml, Sigma, USA) assay. Cells (A549P/A549CisR and H157P/H157CisR) were seeded on 96-well plates (7×10^3^ cells/well) and treated with various concentrations of cisplatin for 48 hours. MTT test was then performed and absorbance at 490 nm was measured. Cell viability was calculated using the formula: OD sample/OD blank control × 100. Triplicate experiments were performed and average values with mean ± SEM were represented.

### Oil Red staining

Parental and cisplatin-resistant cells were grown at an initial density of 10^5^ cells/well in a 24-well plate. After fixation, cells were washed three times and stained with Oil Red O solution (stock solution, 0.7g Oil Red O powder dissolved in 200 ml isopropanol). Working solution was prepared by adding 6 parts stock to 4 parts dH_2_O, mixing and letting sit at room temperature for 20 minutes. Cells were washed to remove unbound staining. The positively stained cells were counted under the microscope.

### Migration test

Tumor cells (A549P/A549CisR and H157P/H157CisR, either vehicle or cerulenin treated, or inhibitor treated, 1 × 10^4^, in no serum containing media) were placed in upper chamber of transwell plates and migration to lower chamber (containing 10% FBS containing media) was analyzed. Migrated cells at the end of 24 hours of incubation were visualized by staining with a crystal violet solution and counted under a microscope. Three independent experiments (with triplicates) were done and average numbers of positively stained cells in three randomly picked areas were presented in quantitation.

### Development of FASN knocked down cell lines by lentiviral transduction

Lentivirus constructs carrying either FASNsiRNA, sc sequence (pLenti-II vector, Addgene) were transfected into 293T cells with a mixture of pLent-II-FASNsiRNA (or sc), psPAX2 (virus-packaging plasmid), and pMD_2_G (envelope plasmid) (4:3:2 ratio) using PolyFect Transfection reagent (Qiagen, Valencia, CA). After infection procedure, the media containing the virus was replaced with normal culture media, and maintained under normal cell culture conditions. The FASN knocked down and sc cells were selected by adding puromycin (2 μg/ml) (Sigma) into the culture. After selection, cells were maintained in media containing 0.1 μg/ml puromycin.

### *In vivo* xenograft studies

The luciferase tagged H157P and H157CisR cells (1 × 10^6^) that were obtained by transfection of luciferase reporter gene and selection procedure were orthotopically injected (1 × 10^6^ cells in media with Matrigel, 1:1 ratio in volume) into 8 week old female nude mice (NCI) (6 mice with H157P cells and 14 mice with H157CisR cells, total 20 mice). Tumor development and volumes were monitored once a week by *In Vivo* Imaging System (IVIS). When luminescence reached 5 × 10^5^ to 1 × 10^6^ radiance (p/sec/cm^2^/sr), mice were divided into two groups. The test group mice (n=7) were i.p. injected with cerulenin (15 mg/kg) and the control group mice (n=7) were injected with vehicle (20% DMSO) for 9 days. Tumor growth and metastasis was monitored during and at the end of treatment. At 3 days after last injection of cerulenin/vehicle, mice were sacrificed and tumor tissues were processed for staining. All animal studies were performed under the supervision and guidelines of the University of Rochester Medical Center‘s Animal Care and Use Committee.

### Histology and immunohistochemistry

Tissues obtained were fixed in 10% (v/v) formaldehyde in PBS, embedded in paraffin, and cut into 5-μm sections. Tumor tissue sections were deparaffinized in xylene solution, rehydrated, and immunostained with the IHC kit (Santa Cruz, Santa Cruz, CA). Antibodies of FASN (Abgent, San Diego, CA) (1:250), Ki67 (Abcam, Cambridge, MA) (1:250), N-Cad (1:200), TGF-β1 (1:150), MMP9 (1:200), and vimentin (Cell Signaling, Danvers, MA) (1:250) were applied in staining. For Ki67 staining, the antigen retrieval process was performed in 10 mM Citric buffer, pH 6.0 for 20 minutes using a cooker prior to staining. After staining, tissues were counterstained by Hematoxylin. The average numbers of positively stained cells were obtained from careful counting of randomly chosen 3 different fields.

### RNA extraction and quantitative real-time PCR (qPCR) analysis

Total RNA (1 μg) was subjected to reverse transcription using Superscript III transcriptase (Invitrogen). qPCR was conducted using the appropriate primers and a Bio-Rad CFX96 system with SYBR green to determine the mRNA expression levels of genes of interest. Expression levels were normalized to GAPDH level.

### Western blot analysis

Cells were lysed in RIPA buffer (50 mM Tris-Cl at pH 7.5, 150 mM NaCl, 1% NP-40, 0.5% sodium deoxycholate, 1 mM EDTA, 1 μg/mL leupeptin, 1 μg/mL aprotinin, 0.2 mM PMSF). Proteins (20-40 μg) were separated on 8–10% SDS/PAGE gel and then transferred onto PVDF membranes (Millipore, Billerica, MA). After blocking procedure, membranes were incubated with primary antibodies (1:1000), HRP-conjugated secondary antibodies (1:5000), and visualized in Imager (Bio-Rad) using ECL system (Thermo Fisher Scientific, Rochester, NY). Antibodies of TGF-β1, FASN, and TWIST were from Gene Tex (Irvine, CA) and the MMP9, and EGR1 antibodies were purchased from Abgent (San Diego, CA). Antibodies of vimentin, E-Cad, and N-cad were from Cell Signaling (Danvers, MA). The GAPDH antibody was purchased from Abcam (Cambridge, UK).

### Statistics

The data were presented as the mean± SEM. Differences in mean values between two groups were analyzed by two-tailed Student's *t* test. *p* ≤ 0.05 was considered statistically significant.

## SUPPLEMENTARY FIGURE


