# Supplementary material for: A FASN-TGF-β1-FASN regulatory loop contributes to high EMT/metastatic potential of cisplatin-resistant non-small cell lung cancer
Source: Oncotarget. 2016 Jul 25;7(34):55543–54. doi: 10.18632/oncotarget.10837 (PMC5342435; doi:10.18632/oncotarget.10837)
Supplement: Supplementary file 1 [file oncotarget-07-55543-s001.pdf]

A FASN-TGF-β1-FASN regulatory loop contributes to high EMT/metastatic potential of cisplatin-resistant non-small cell lung cancer

SUPPLEMENTARY FIGURE

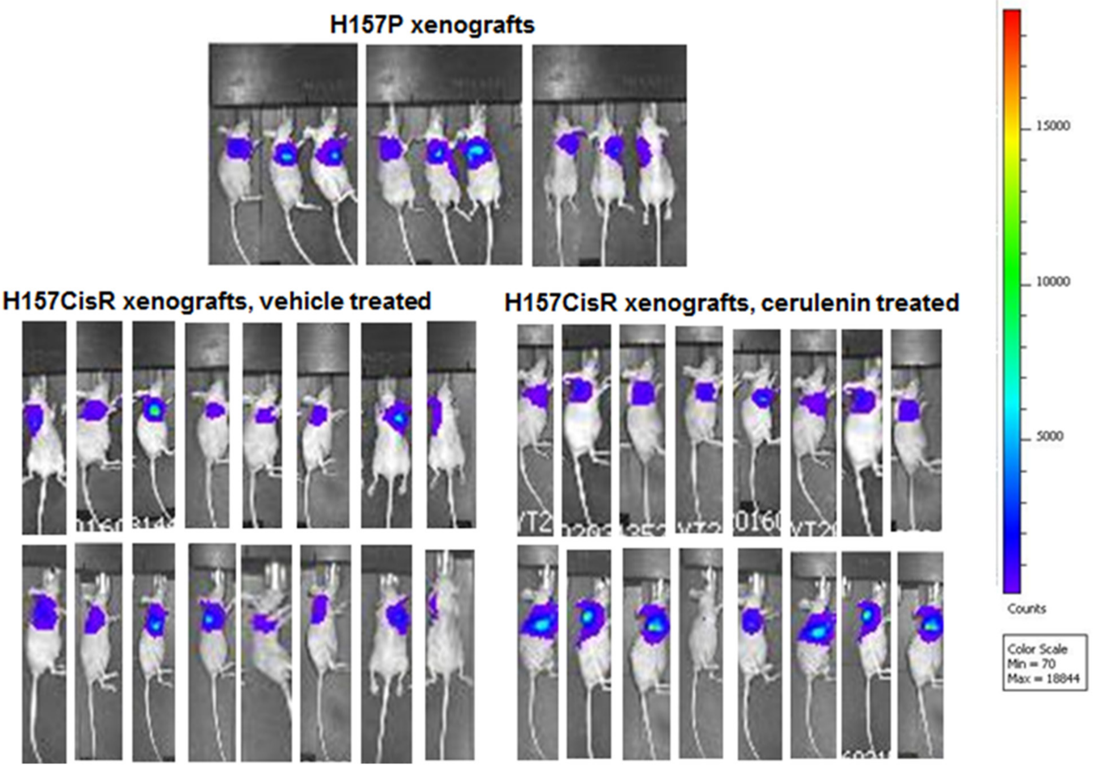

Supplementary Figure S1: IVIS image of mice bearing H157P cell-derived and H157CisR cell-derived (treated with either vehicle or cerulenin) tumors at 3 weeks of treatment.
